# Glycyrrhizic Acid Alleviates Semen Strychni-Induced Neurotoxicity Through the Inhibition of HMGB1 Phosphorylation and Inflammatory Responses

**DOI:** 10.1007/s11481-024-10128-8

**Published:** 2024-05-21

**Authors:** Changwei Yu, Yalan Xiang, Min Zhang, Jing Wen, Xiaoyu Duan, Lu Wang, Gongying Deng, Pingfei Fang

**Affiliations:** 1 Department of Pharmacy, the Second Xiangya Hospital, Central South University, Changsha, 410011 China; 2 Department of Pharmacy, Hunan Provincial Maternal and Child Health Care Hospital, Changsha, 410008 China; 3 Institute of Clinical Pharmacy, Central South University, Changsha, 410011 China; 4 Department of Pharmacy, the Third Hospital of Changsha, Changsha, 410015 China

**Keywords:** Semen strychni, Neurotoxicity, Glycyrrhizic acid, HMGB1, Phosphorylation

## Abstract

The neurotoxicity of Semen Strychni has been reported recently in several clinical cases. Therefore, this study was conducted to investigate the role of HMGB1 in a model of neurotoxicity induced by Semen Strychni and to assess the potential alleviating effects of glycyrrhizic acid (GA), which is associated with the regulation of HMGB1 release. Forty-eight SD rats were intraperitoneally injected with Semen Strychni extract (175 mg/kg), followed by oral administration of GA (50 mg/kg) for four days. After treatment of SS and GA, neuronal degeneration, apoptosis, and necrosis were observed via histopathological examination. Inflammatory cytokines (TNF-α and IL-1β), neurotransmitter associated enzymes (MAO and AChE), serum HMGB1, nuclear and cytoplasmic HMGB1/ph-HMGB1, and the interaction between PP2A, PKC, and HMGB1 were evaluated. The influence of the MAPK pathway was also examined. As a result, this neurotoxicity was characterized by neuronal degeneration and apoptosis, the induction of pro-inflammatory cytokines, and a reduction in neurotransmitter-metabolizing enzymes. In contrast, GA treatment significantly ameliorated the abovementioned effects and alleviated nerve injury. Furthermore, Semen Strychni promoted HMGB1 phosphorylation and its translocation between the nucleus and cytoplasm, thereby activating the NF-κB and MAPK pathways, initiating various inflammatory responses. Our experiments demonstrated that GA could partially reverse these effects. In summary, GA acid alleviated Semen Strychni-induced neurotoxicity, possibly by inhibiting HMGB1 phosphorylation and preventing its release from the cell.

## Introduction

Semen Strychni is derived from the dried mature seeds of the medicinal plant Strychnos nux-vomica. This plant has been found to possess various pharmacological activities, including anti-tumor, anti-inflammatory, immunosuppression, and nerve excitation (Zheng et al. 2012; Agrawal et al. 2011a, b; Rao and Prasad M N 2013). However, excessive or prolonged use of this plant can result in multiple organ damage, particularly neurotoxicity, which limits its clinical application (Chen et al. 2014; Philippe et al. 2004). Studies have identified that brucine and strychnine are its chief bioactive and toxic compounds (Wu et al. 2007). The overdose of strychnine can cause the over-limit inhibition of the cerebral cortex and involuntary contractions of respiratory muscles, ultimately resulting in cardiac arrest or asphyxia (Philippe et al. 2004). As for brucine, its toxic dose can block neuromuscular transmission (Philippe et al. 2004). Studies have demonstrated that rats poisoned with Semen Strychni exhibited severe oxidative stress and brain inflammation (Hou et al. 2017; Li et al. 2018; Shi and Hou 2017). Therefore, it is crucial to elucidate the neurotoxicity mechanism of Semen Strychni and find an effective antidote.

Relevant studies have proven that neuroinflammation after brain injury is linked to high-mobility group box 1 (HMGB1), including, among others, epilepsy (Fang et al. 2012; Ravizza et al. 2018), cerebral ischemia (Kim S W, Jin Y, Shin et al. 2012), and neuropathy (Nishida et al. 2016). HMGB1 is a highly conserved non-histone nuclear protein involved in regulating gene transcription and stabilizing nucleosome structure by binding to DNA. Under normal physiological conditions, HMGB1 primarily exists in the nucleus and functions as a nuclear-binding protein. However, when cells or tissues are injured, HMGB1 is released from the nucleus to the outside of the cell, where it tends to become a neuroinflammatory factor (Kang et al. 2014). Its principal mechanism is to activate the NF-κB and mitogen-activated protein kinase (MAPK) pathways by binding to Toll-like receptors (TLRs) and receptors for advanced glycation end products, further inducing the secretion of inflammatory cytokines, thereby enhancing neuroinflammation (Kim S W, Lim C M, Kim et al. 2011; Liu et al. 2019; Paudel Y N et al. 2020). To sum up, HMGB1’s role depends on its position. Luo (Youn and Shin 2006) noted that the level of HMGB1 was up-regulated and accumulated in serum in kainic acid-induced seizure mice. In addition, there is a nuclear localization signal located in the HMGB1 sequence, where the serine phosphorylation state determines the subcellular localization of HMGB1. When serine residues are hyperphosphorylated, HMGB1 is more easily translocated from the nucleus to the cytoplasm. Therefore, HMGB1 phosphorylation is considered critical for its extracellular release (Youn and Shin 2006). Studies have reported that protein kinase C (PKC) (Zhang and Tang 2008; Kang et al. 2009) can phosphorylate HMGB1 and promote its release from the cell, while protein phosphatase 2 A (PP2A) can dephosphorylate the phosphorylated HMGB, thereby reversing this trend (Taira et al. 2013). In conclusion, Semen Strychni administration with a toxic dose can lead to massive necrosis of neurons. HMGB1 may be a critical cytokine in inducing neurotoxicity and inflammation, and phosphorylation is the key extracellular form of HMGB1 (Shi and Hou 2017; Shichita et al. 2012).

Glycyrrhizic acid (GA)—also known as glycyrrhizin—is one of the main active components of licorice. As a natural anti-inflammatory and antiviral triterpenoid, GA is extensively used clinically, mainly for treating acute and chronic hepatitis, viral hepatitis, and other liver diseases, as well as a cellular immune regulator (Liu Y F, Gao et al. 2012). It exerts anti-inflammatory, antioxidant, and other neuroprotective pharmacological effects (Chen I C et al. 2018; Wang et al. 2013, 2021). Moreover, we found that the pharmacological effects of GA in neurological diseases or brain injury are closely related to the molecule HMGB1, which accumulated in the serum of epilepsy model mice but down-regulated after GA administration (Luo et al. 2014). Besides, the improvement in intracerebral hemorrhage injury is linked to HMGB1 protein (Huang et al. 2013). Studies have suggested that GA can improve injury and neurological dysfunction by blocking the inflammation triggered by microglia and the HMGB1-TLR4 signaling pathway after brain injury, thereby exerting neuroprotective effects (Sun et al. 2018a, b; Zhang et al. 2014). Our previous experiments demonstrated that the toxicity of Semen Strychni significantly reduced after the use of licorice (Zhang et al. 2018). Therefore, whether GA, the chief active component of licorice, can alleviate the severe neurotoxicity caused by Semen Strychni and its underlying mechanism warrants further examination.

In this study, we explored the neurotoxicity of Semen Strychni mediated by HMGB1 and then sought possible ways to alleviate the toxicity by administration of GA, an HMGB1 inhibitor. This study aims to provide a reference for improving the clinical safety of Semen Strychni.

## Materials and Methods

### Plant Material

Semen Strychni (batch number 201,022,891) was obtained from SanXiang Co. Ltd. (Changsha, China). All herbs were authenticated by Professor Yu-hua Wang (Department of Pharmacy, the Second Xiangya Hospital, Central South University). Voucher specimens of the two herbs were deposited at the Department of Pharmacy, the Second Xiangya Hospital.

### Extraction of Herbs

After the raw Semen Strychni seeds were crushed, a proper amount of powder was taken and soaked in 75% acidic ethanol (pH = 5, 1:12, W/V) for 12 h and then heated to reflux thrice, each for 1 h. The extract was filtered using a 0.45 μm microporous filter membrane under hot conditions. Then, the filtrate was combined and concentrated by a rotary evaporator until all ethanol was volatilized. The filtrate pH was adjusted to 6.5 with 0.5 mol/L NaOH. The extract was mixed with 1% carboxymethylcellulose sodium (CMC-Na) solution and water to prepare 50 mg raw Semen Strychni/0.5%CMC-Na mL. The composition of the Semen Strychni extract was detected by high-performance liquid chromatography coupled with tandem mass spectrometry and ultraviolet detector (HPLC-UV-MS).

### Animals Experiments

The experimental animals were SPF-grade male SD rats purchased from Hunan SJA Laboratory Animal Co., Ltd., with the license number SCXY (Xiang) 2019-0004. The rats were raised in the Department of Laboratory Animal Science of Central South University, and the experimental unit’s animal use license number is SYXK (Xiang) 2015-0017. This study was reviewed and approved by the Laboratory Animal Management Committee of the Department of Laboratory Animal Science of Central South University (acceptance number: 2020sydw0821). Forty-eight rats were used to assess the hippocampal injury induced by Semen Strychni and randomly divided into four groups (*n* = 12) as follows:

Blank group (Control): Rats were intraperitoneally injected with 0.5% CMC-Na solution with a volume of 3.5 mL/kg. After the injection, 0.5% CMC-Na solution was given by intragastric administration at a volume of 5 mL/kg.

Strychni poisoning group (SS): Rats were intraperitoneally injected with the extract of Semen Strychni (0.175 g/kg). Immediately after the injection, 0.5% CMC-Na solution was given by intragastric administration with a volume of 5.0 mL/kg.

Low-dose GA detoxification group (SS + LGA): Rats were intraperitoneally injected with the extract of Semen Strychni (0.175 g/kg), and GA (75 g/kg) was then given by gavage.

High-dose GA detoxification group (SS + HGA): Rats were intraperitoneally injected with the extract of Semen Strychni (0.175 g/kg), and GA (150 g/kg) was then given by gavage.

Semen Strychni and GA were given for four days.

### Sample Collection

At the time of dissection, the blood was collected from the heart and then centrifuged (4 °C, 3500 rpm) for 10 min. The supernatant was extracted to obtain serum. The wholly separated brain tissues of rats were randomly selected, soaked, and cleaned with normal saline several times and then put into a centrifuge tube with paraformaldehyde.

### Histopathological Examination

#### Nissl Staining

The rat brains were fixed with 4% paraformaldehyde and embedded in paraffin. The paraffin was then sectioned, dewaxed, and stained with toluidine blue after hydration. The sections were placed in xylene solution for 5 min to make them transparent and then mounted with neutral gum. Images of the rat hippocampus were collected under a microscope and analyzed.

#### TUNEL Staining

After paraffin embedding, sectioning, dewaxing, and other steps in the same way as above, proteinase K working solution was added dropwise for antigen retrieval, followed by membrane rupture, labeling with TdT: dUTP at 1:9, DAPI counterstaining, and anti-fluorescence quenching. After mounting with an anti-mounting medium, the tissue sections were observed under a fluorescence microscope, and images were collected.

#### FJB Staining

After dewaxing and hydrating the paraffin sections, FJB dilution was prepared according to the ratio of 50% glacial acetic acid to 1:400 for staining. The nuclei were stained with DAPI and then placed in xylene solution to seal the sections with transparent neutral resin. The tissue sections were then observed under a fluorescence microscope, and images were collected.

### ELISA

The collected blood was centrifuged at 2500 rpm for 15 min at 4 °C, and serum was collected for ELISA detection. AChE, MAO, HMGB1, TNF-α, and IL-1β were quantitatively detected according to the instructions.

### Western Blotting

Brain hippocampus tissues were centrifuged at 12,000 g for 10 min following homogenization in RIPA lysis buffer (1:10, w/v). The super-natants were collected. The whole protein samples were obtained by this method. The nucleus and cytoplasm protein were obtained by using the nucleus and cytoplasmic extraction kit. (Boster Biological Technology Co.,Ltd) All protein samples were determined by BCA protein assay kit. Make appropriate dilution of serum and tissue samples based on the protein concentration. Then the samples were mixed with 5× loading buffer, then were boiled at 100◦C for 5 min and stored at − 70◦C until further analysis.

Proteins were run on 10% or 12% polyacrylamide gels and transferred to polyvinylidene fluoride (PVDF) membranes. The membranes were blocked with 5% BSA for 60–90 min at room temperature and incubated with antigen-specific primary antibodies overnight at 4 °C. Blots were then incubated with species-specific secondary antibodies for 60 min at room temperature. Proteins were visualized by incubation with a chemiluminescent substrate kit. The primary antibodies used in this study were anti-HMGB1 (10829-1-AP, Proteintech, USA), anti-p-serine (ab9332, Abcam, UK), anti-NF-κB (10745-1-AP, Proteintech, USA), anti-p-38 (14064-1-AP, Proteintech, USA), anti-PP2A (13482-1-AP, Proteintech, USA), and anti-PKC (21991-1-AP, Proteintech, USA).

### Immunofluorescent Staining

The paraffin sections were dewaxed and hydrated, and autofluorescence quenchers were added dropwise to the tissue sections after antigen retrieval. BSA solution was added dropwise for blocking. The primary antibody was added overnight at 4 °C. The secondary antibody was added and reacted at room temperature for 50 min. DAPI staining solution was added dropwise on the tissue to counterstain the nuclei. Slides were mounted with an anti-fluorescence quenching mounting medium. The tissue sections were then observed under a fluorescence microscope, and images were collected.

### Co-immunoprecipitation

Total protein was extracted from each group and stored at − 80 °C. HMGB1 antibody was added to the protein supernatant, and the antigen–antibody mixture was shaken slowly at 4 °C and incubated overnight. Protein A/G agarose beads were taken, and IP lysis buffer was added to them and centrifuged at 3000 rpm to retain the pellet. The cell lysate incubated with the antibody overnight was added to the pretreated protein A/G agarose beads to couple the antibody to the protein A/G agarose. After co-immunoprecipitation, centrifugation was performed at 3000 rpm for 3 min at 4 °C, the supernatant was aspirated, and the pellet was collected. Expression of p-HMGB1 with PP2A and PKC was assessed using western blotting analysis.

### Statistical Analysis

SPSS 18.0 software was used for statistical analysis of data. Data are presented as mean ± standard error (mean ± SEM). One-way analysis of variance (ANOVA) was used for comparison between multiple groups, and the LSD test was used for comparison between two groups. When the p-value was less than 0.05, the difference was regarded as statistically significant. The graphing software used was GraphPad Prism 8.0.

## Results

### Glycyrrhizic acid Relieves Neuronal Damage Caused by Semen Strychni

**Fig. 1:**
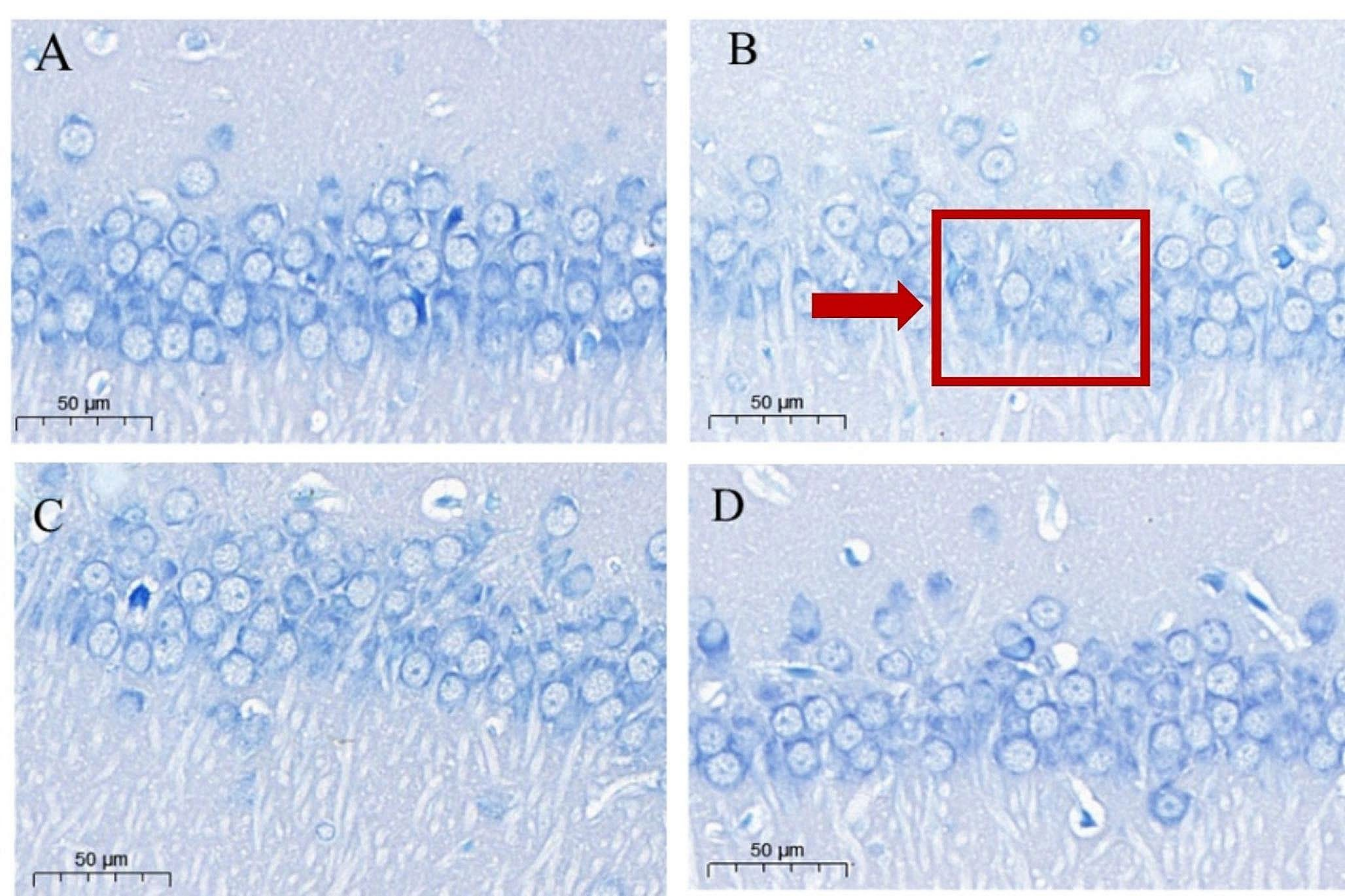
Effects of Glycyrrhizic acid and Semen Strychni on Nissl staining in the CA1 area of rat hippocampus. Representative photographs of Nissl staining were obtained from the (**A**) Control group; (**B**) SS group; (**C**) SS + LGA group; (**D**) SS + HGA group

Figure 1 depicts the results of Nissl staining of neurons in the CA1 region of the hippocampus of rats in each group. Compared with the Control group, the neurons in the SS group were greatly reduced, the cell integrity was lost, the distribution of Nissl bodies was uneven, and the staining was blurred (Fig. 1-B), indicating that the Semen Strychni caused the hippocampal neuronal damage. After GA treatment, the neuronal damage in the SS + LGA group (Fig. 1-C) and the SS + HGA group (Fig. 1-D) was relieved. This showed that the number of Nissl bodies increased, the staining was darker, and the neurons were arranged more closely.

**Fig. 2:**
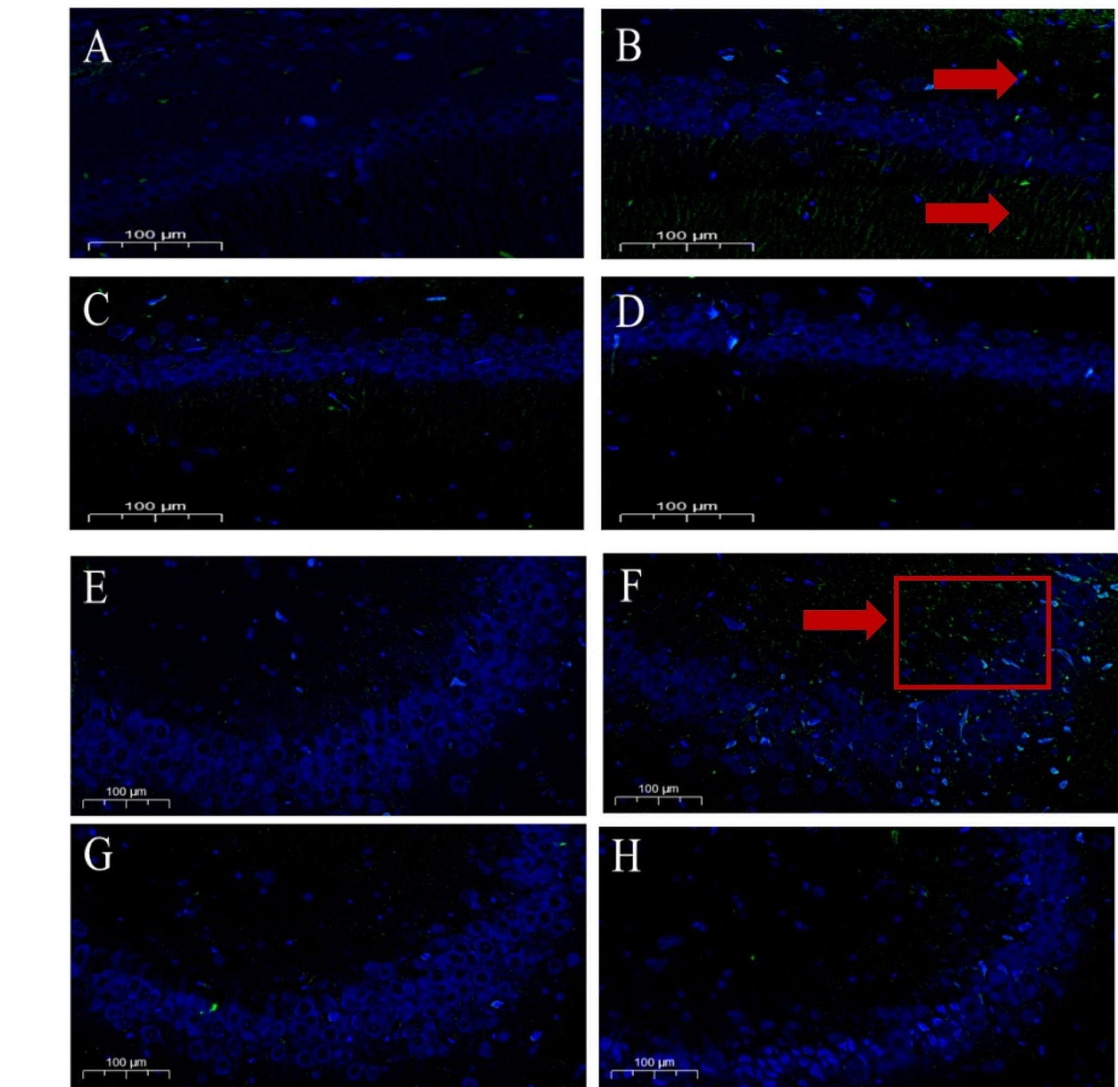
Effects of Glycyrrhizic acid and Semen Strychni on Nissl staining in the CA1 and CA3 areas of rat hippocampus. Representative photographs of FJB staining were obtained from the (**A**) and (**E**) Control group; (**B**) and (**F**) SS group; (**C**) and (**G**)SS + LGA group; (**D**) and (**H**) SS + HGA group

FJB histofluorescence staining is a sensitive technique to detect neuronal degeneration. As depicted in Figs. 2-A and 2-E, almost no degeneration and necrosis were found in the hippocampal CA1 and CA3 regions of the rats in the Control group. Compared with the control group, the number of FJB-positive cells in the Semen Strychni neurotoxicity model group increased, especially in the CA3 area (Fig.  2-F). Very few were observed in the CA1 and CA3 regions of the SS + LGA group (Figs. 2-C and 2-G) and the SS + HGA group (Figs. 2-D and 2-H). No significant difference existed in FJB-positive neurons compared with the Control group, indicating that GA exerts an ameliorating effect on nerve cell necrosis.

**Fig. 3:**
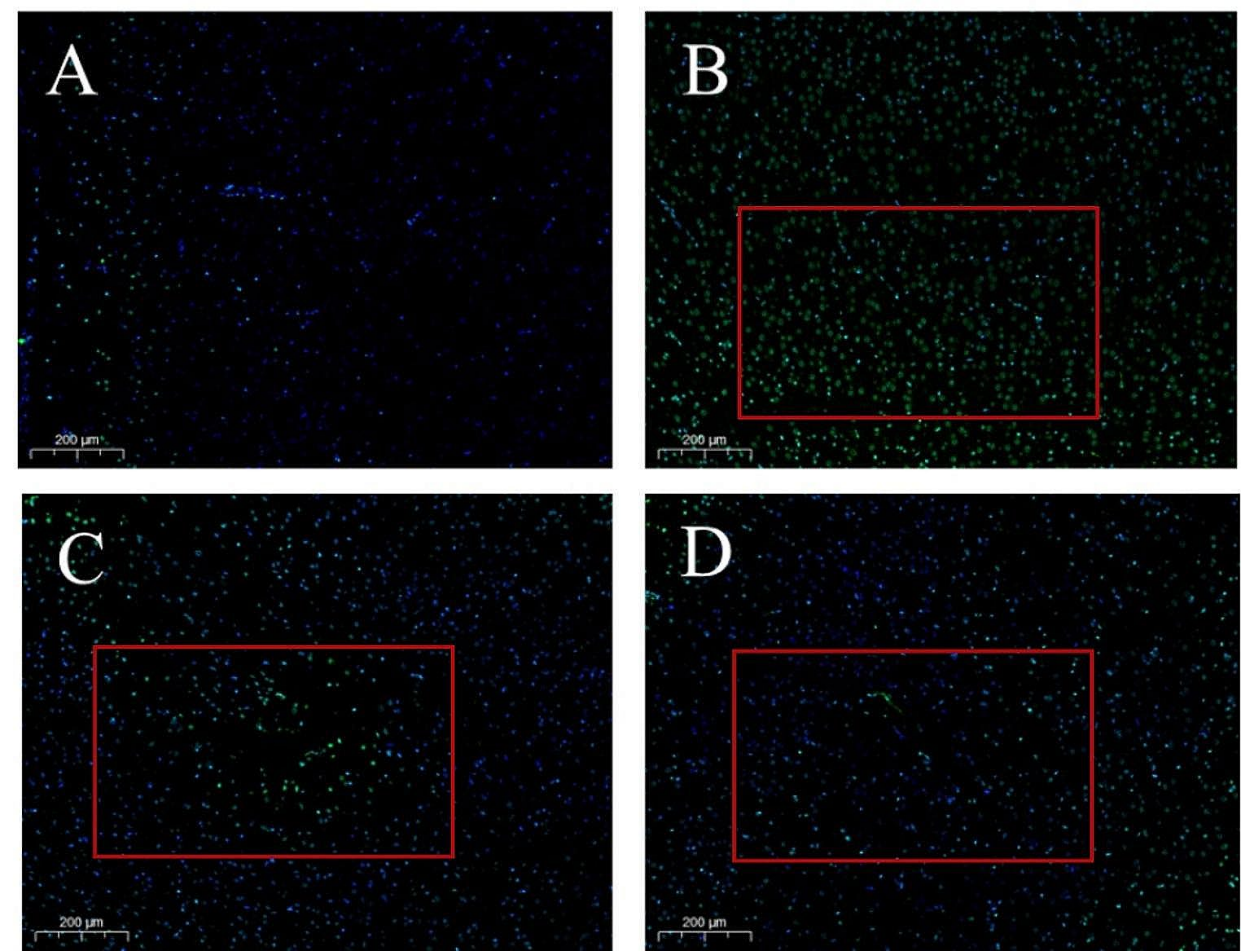
Effects of Glycyrrhizic acid and Semen Strychni on TUNEL staining of rat cortex. Representative photographs of TUNEL staining were obtained from the (**A**) Control group; (**B**) SS group; (**C**) SS + LGA group; (**D**) SS + HGA group

The results of TUNEL staining are presented in Fig. 3, in which there are a small number of TUNEL-positive cells in the cortex of the rats in the Control group, while the number of positive cells in the SS group is significantly increased, and many apoptotic cells are distributed. The overall distribution of apoptotic cells in SS + LGA and SS + HGA groups was less than that in the SS group, suggesting that GA exerts a protective effect on Semen Strychni-induced neuronal apoptosis.

### Glycyrrhizic acid Relieves Disturbance of Neurometabolic Enzyme Levels Caused by Semen Strychni

**Fig. 4:**
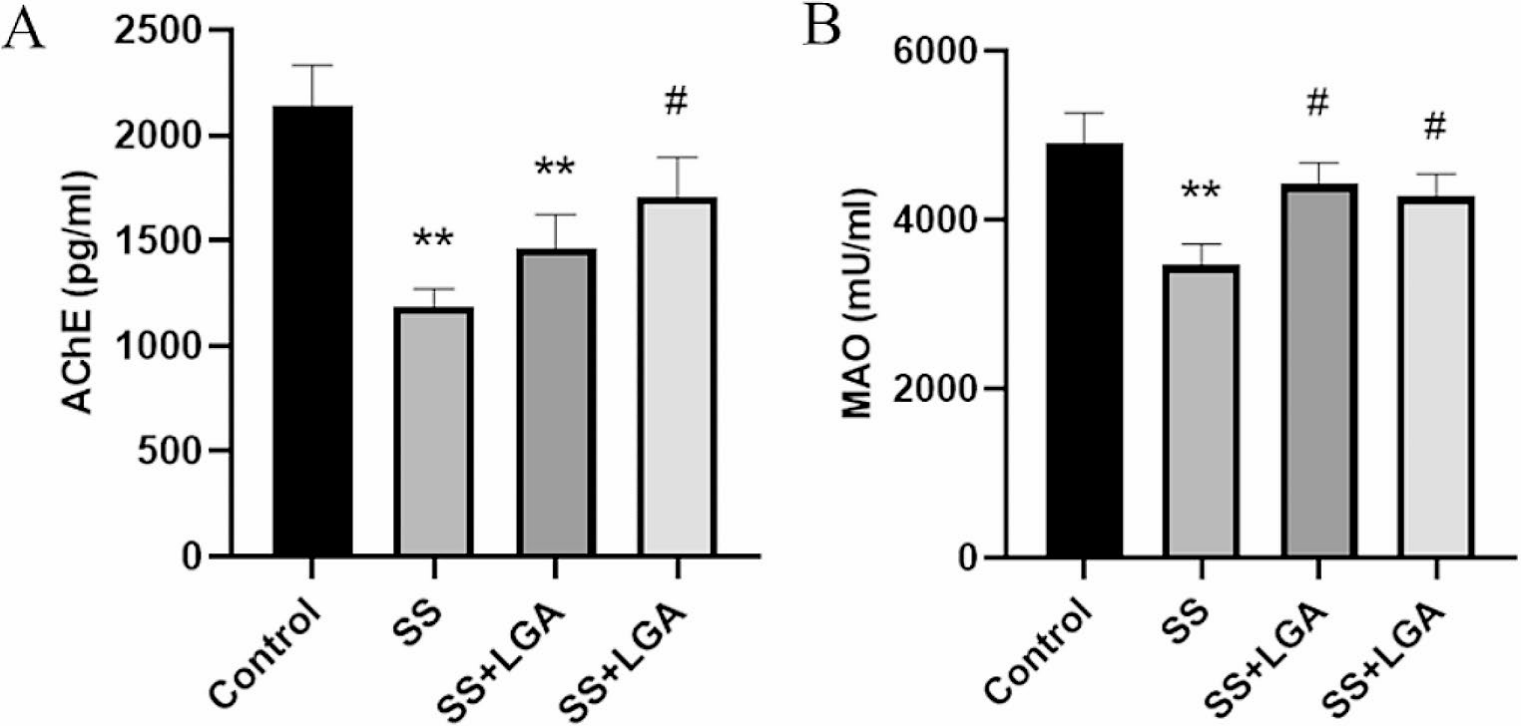
The AChE (**A**) and MAO (**B**) levels of different groups in the serum. Data are presented as means ± SEM (*n* = 8–10). *p < 0.05, **p < 0.01 vs. the Control group. #*p* < 0.05, ##*p* < 0.01 vs. the SS group

Figure 4 depicts the levels of AChE and MAO in the serum of rats in each group. After Semen Strychni administration, the levels of AChE and MAO in the serum of the rats in the SS group were significantly lower than those in the Control group (*p* < 0.01). High doses of GA significantly up-regulated AChE levels (*p* < 0.05). As for MAO, both high and low doses of GA significantly increased the level of MAO (*p* < 0.05), demonstrating that GA could effectively regulate the neurotransmitter metabolism disorder caused by Semen Strychni and make the levels of related enzymes return to normal.

### Glycyrrhizic Acid Inhibits HMGB1 Release and Nucleocytoplasmic Translocation

**Fig. 5:**
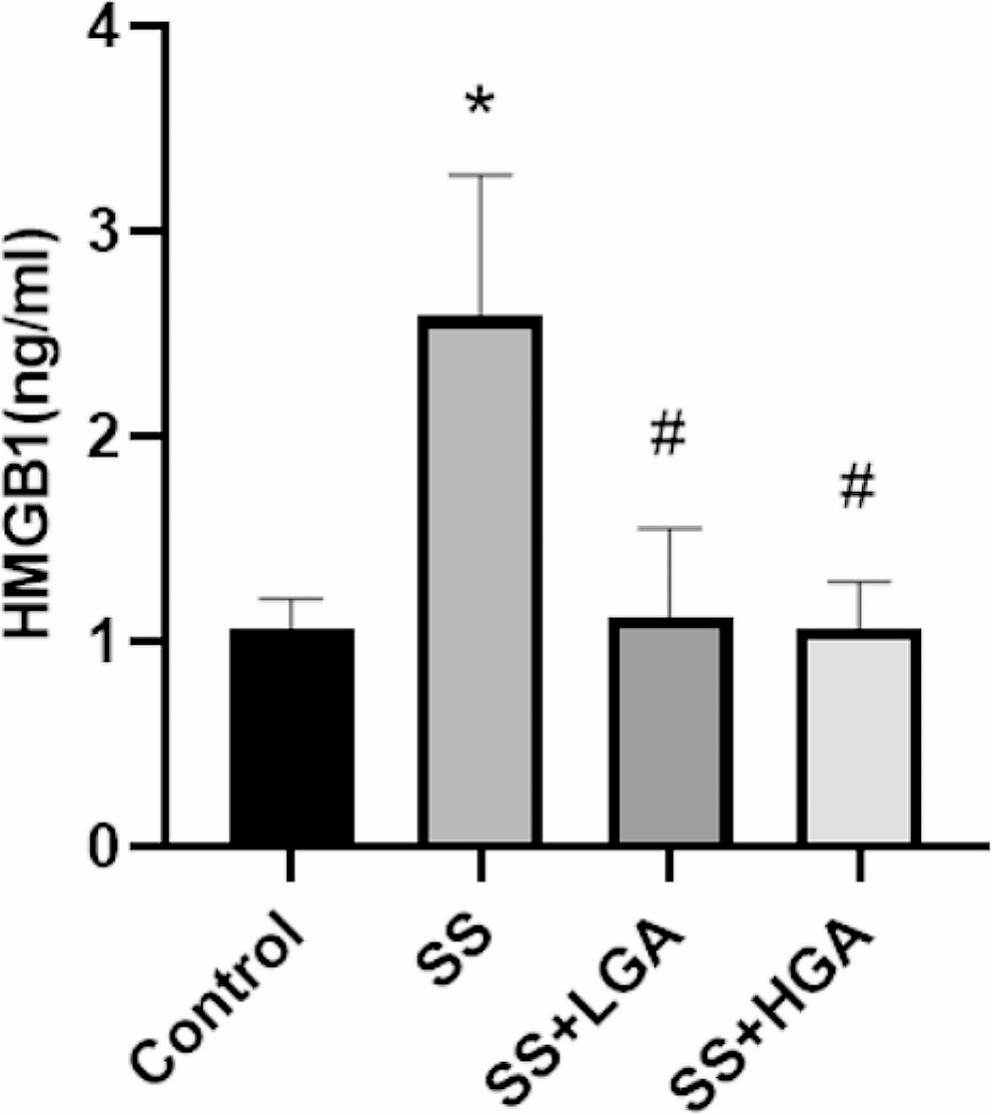
The HMGB1 levels of different groups in the serum. Data are presented as means ± SEM (*n* = 8–10). *p < 0.05 vs. the Control group. #*p* < 0.05 vs. the SS group

**Fig. 6:**
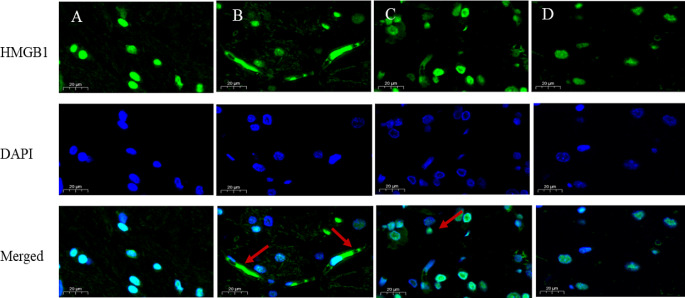
Subcellular localization of HMGB1 in rat brain. Representative photographs of IF (HMGB1) were obtained from the (**A**) Control group; (**B**) SS group; (**C**) SS + LGA group; (**D**) SS + HGA group

**Fig. 7:**
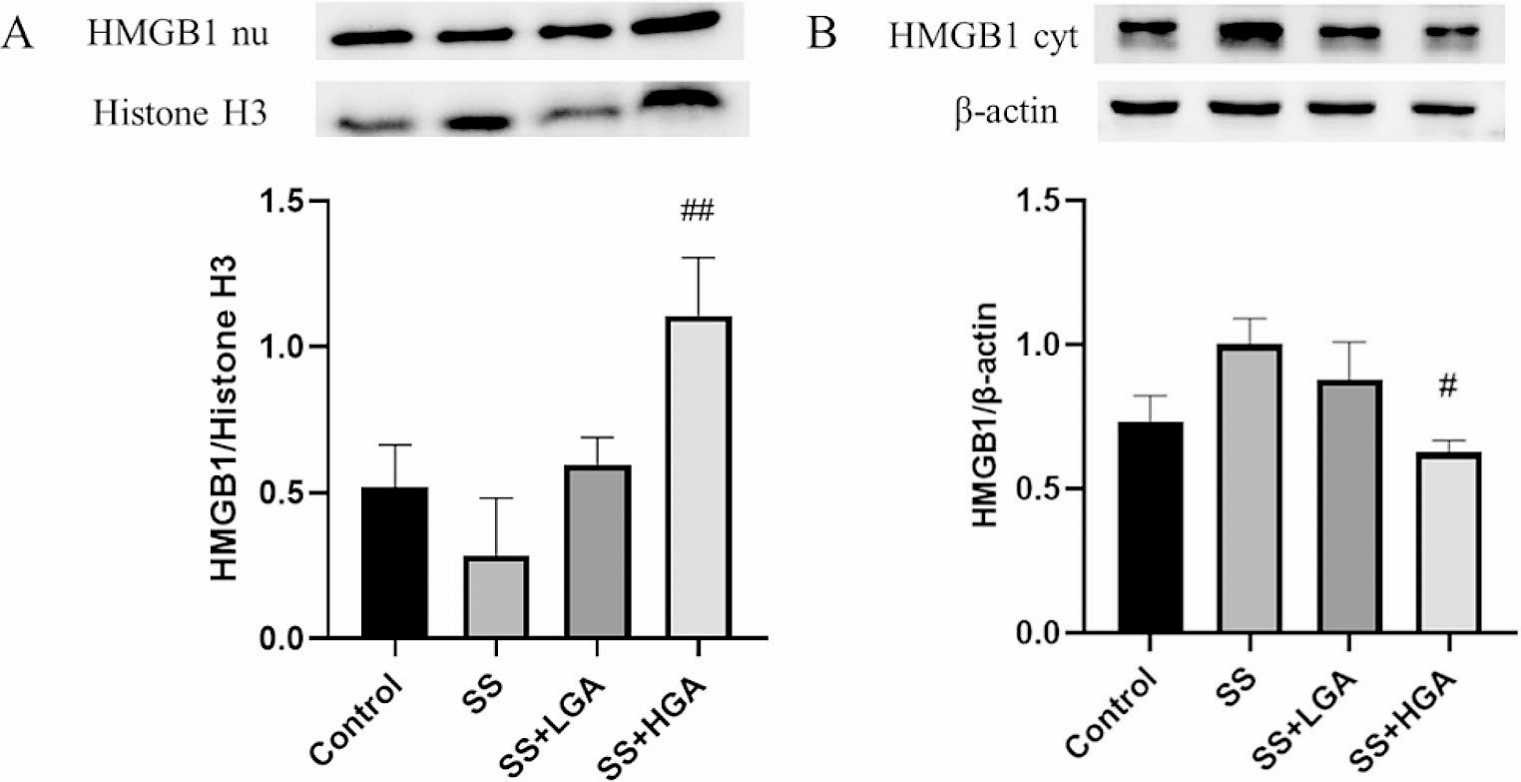
Protein expression of HMGB1 in the nucleus and cytoplasm. (**A**) Expression of HMGB1 protein in the nucleus. (**B**) Expression of HMGB1 protein in the cytoplasm. The blot shown is representative of three experiments with similar results. Data are presented as means ± SEM (*n* = 3). *p < 0.05, **p < 0.01 vs. the Control group. #*p* < 0.05, ##*p* < 0.01 vs. the SS group

As a downstream mediator of systemic inflammation, HMGB1 is believed to play a crucial role in brain injury (Li et al. 2020), and its location is more critical. Severe inflammation occurs when HMGB1 is released from the nucleus to the cytoplasm and serum. Figure 5 depicts the level of HMGB1 in serum, which is the amount of HMGB1 that is released extracellularly. Compared with the Control group, the extracellular HMGB1 level in the SS group was significantly increased (*p* < 0.05), which indicated that Semen Strychni could increase the release of HMGB1. After GA administration, HMGB1 recovered to the level of the blank group (*p* < 0.05). In the Control group, Fig. 6-A shows that HMGB1 is distributed in the nucleus, and there is almost no positive signal outside the nucleus. In the SS group (Fig. 6-B), the primary distribution of HMGB1 is no longer limited to the nucleus but has a significant increase in the cytoplasm. Compared with the SS group, the cytoplasmic distribution of HMGB1 in the SS + LGA group was improved to a certain extent, while in the SS + HGA group, the inhibitory effect was more evident, and HMGB1 was primarily distributed in the nucleus. Then, we further validated the expression levels of HMGB1 protein in the nucleus and cytoplasm by western blotting. The results presented in Fig. 7 are generally consistent with the immunofluorescence results (The full uncropped Gels and Blots images in supplementary file Fig. 7). The expression of HMGB1 was found to be the lowest in the nucleus of the SS group and highest in the cytoplasm of the SS group. Consistent with the immuno-histochemical results, high-dose GA significantly increased the expression level of HMGB1 in the nucleus (*p* < 0.01) and reduced the cytoplasmic HMGB1 level to a normal level (*p* < 0.01).

### Glycyrrhizic acid regulates the interaction of dephosphorylate and phosphokinase with HMGB1 to inhibit Semen Strychni-induced HMGB1 phosphorylation

**Fig. 8:**
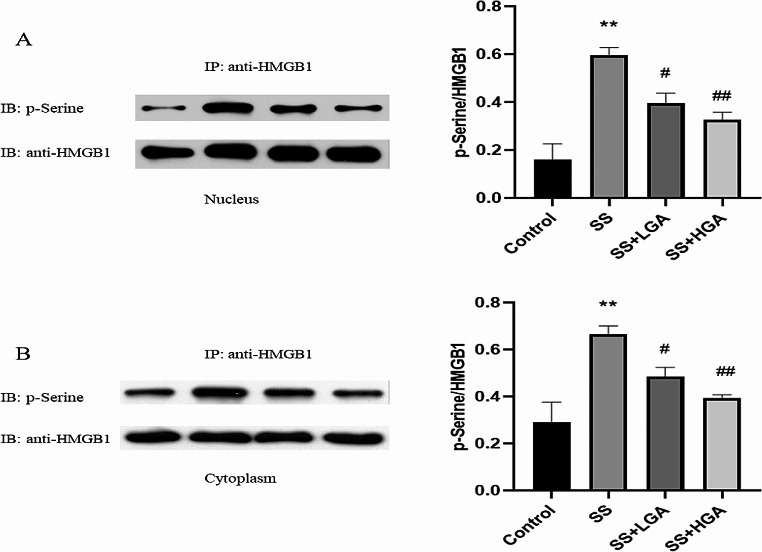
Effects of glycyrrhizic acid and Semen Strychni on levels of HMGB1 phosphorylation. (**A**) Expression of phosphorylated HMGB1 protein in the nucleus. (**B**) Expression of phosphorylated HMGB1 protein in the cytoplasm. Nuclear and cytosol extracts were immunoprecipitated (IP) with an anti-HMGB1 antibody and then immunoblotted (IB) with an anti-p-serine antibody and an anti-HMGB1 antibody. The blot shown is representative of three experiments with similar results. Data are presented as means ± SEM (*n* = 3). *p < 0.05, **p < 0.01 vs. the Control group. #*p* < 0.05, ##*p* < 0.01 vs. the SS group

**Fig. 9:**
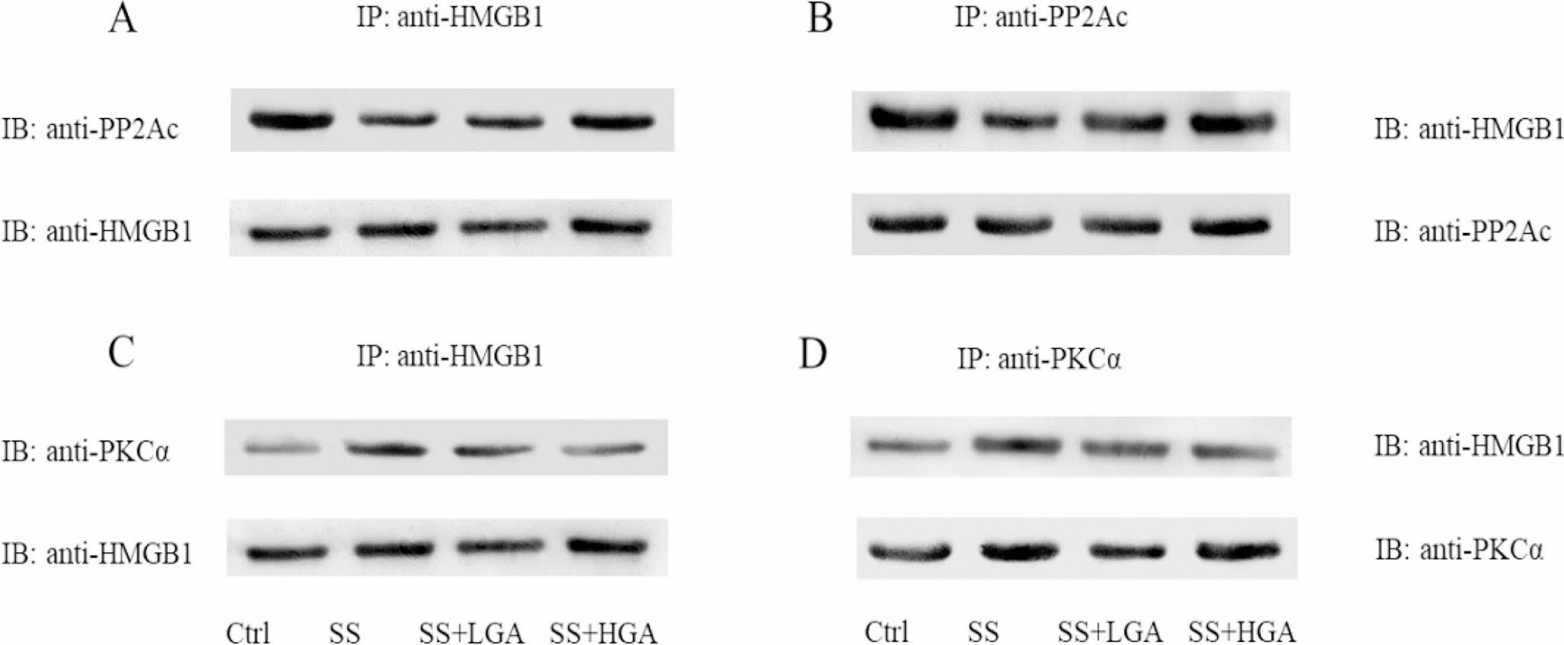
Protein–protein interactions between HMGB1 and PP2A (**A-B**) and between HMGB1 and PKC (**C-D**) were examined by immunoprecipitation-linked immunoblotting. The whole-cell lysates were prepared and immunoprecipitated with anti-HMGB1 antibody and then immunoblotted with anti-PP2Ac antibody (**A**) or immunoprecipitated with anti-PP2Ac antibody and then immunoblotted with anti-HMGB1 antibody (**B**). The whole tissue lysates were prepared and immunoprecipitated with anti-HMGB1 antibody and then immunoblotted with anti-PKCα antibody (**C**) or immunoprecipitated with anti-PKCα antibody and then immunoblotted with anti-HMGB1 antibody (**D**)

When HMGB1 is phosphorylated, it is easier to release from the nucleus. Figs 8-A and 8-B depict the phosphorylation levels of HMGB1 in the nucleus and cytoplasm, respectively. Compared with the Control group, the phosphorylation levels in the nucleus and cytoplasm of the SS group were significantly increased (*p* < 0.01). GA could significantly inhibit the phosphorylation of HMGB1. PP2Ac could dephosphorylate phosphorylated HMGB1 within the nucleus, thereby preventing the release of HMGB1. We used HMGB1 as the bait protein to precipitation to detect the expression of the protein PP2Ac (Fig. 9-A) and then verified the binding between PP2Ac and HMGB1 in reverse (Fig. 9-B). The results showed that the interaction between PP2Ac and HMGB1 was weakest in the SS group. Compared with the SS group, the binding of PP2Ac to HMGB1 was increased in the GA group. In brief, GA inhibited the release of HMGB1 by facilitating the binding between PP2Ac and HMGB1. In contrast to the above, PKCα could phosphorylate HMGB1 and promote its release. Figs. 9-C and 9-D display the results of detecting the binding of HMGB1 to PKCα. The binding of HMGB1 to PKCα was enhanced after Semen Strychni administration; however, this binding was partially blocked by GA (The full uncropped Gels and Blots images in supplementary file Figs. 8 and 9**)**.

### Glycyrrhizic Acid Inhibits the Release of Inflammatory Factors and the Activation of Inflammatory Pathways Induced by Semen Strychnin

**Fig. 10:**
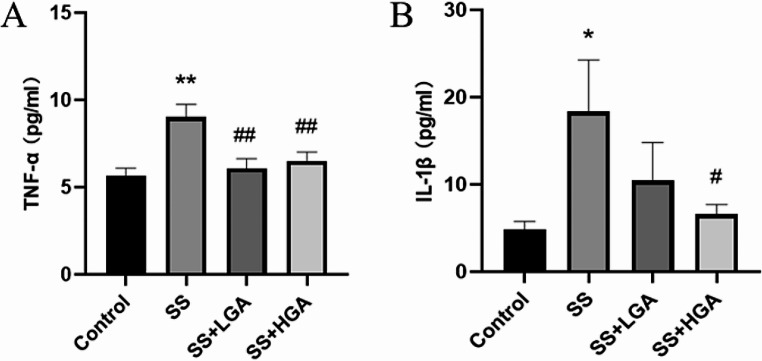
The TNF-α (**A**) and IL-1β (**B**) levels of different groups in the serum. Data are presented as means ± SEM (*n* = 8–10). *p < 0.05, **p < 0.01 vs. the Control group. #*p* < 0.05, ##*p* < 0.01 vs. the SS group

Studies have proven that neuroinflammation plays a key role in central nervous system injury, potentially damaging nerve cells (Yang Q Q, Zhou 2019). Activation of MAPK family members such as TNF-α and IL-1β by HMGB1 induces pro-inflammatory cytokines during injury (Fang et al. 2012). Figure 10 displays the levels of pro-inflammatory factors TNF-α and IL-1β in the serum of rats in each group. In the Control group, the concentrations of TNF-α and IL-1β were both at lower levels. After administration of Semen Strychni, the concentrations of TNF-α and IL-1β in the serum of rats in the SS group were significantly increased (*p* < 0.05), indicating that Semen Strychni could promote the release of inflammatory factors and aggravate the inflammatory response. After GA intervention, TNF-α concentration in SS + LGA and SS + HGA groups was significantly lower than that in the SS group (*p* < 0.01). This indicates that GA could reverse the release of pro-inflammatory factors caused by Semen Strychni and exert an anti-inflammatory effect. In addition, high-dose GA significantly down-regulated IL-1β levels (*p* < 0.05).

**Fig. 11:**
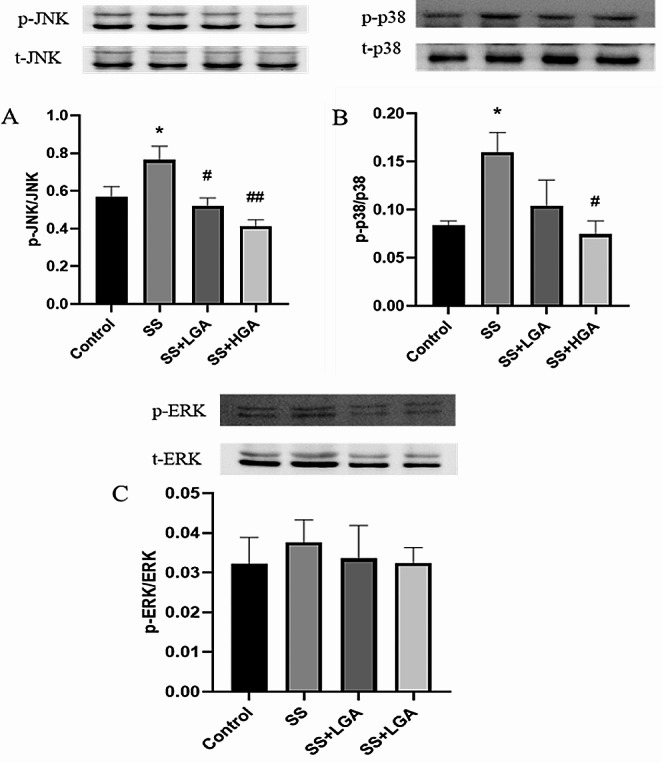
Effects of glycyrrhizic acid and Semen Strychni on activation of the MAPK pathway. The blot shown is representative of three experiments with similar results. Data are presented as means ± SEM (*n* = 3). *p < 0.05, **p < 0.01 vs. the Control group. #*p* < 0.05, ##*p* < 0.01 vs. the SS group

**Fig. 12:**
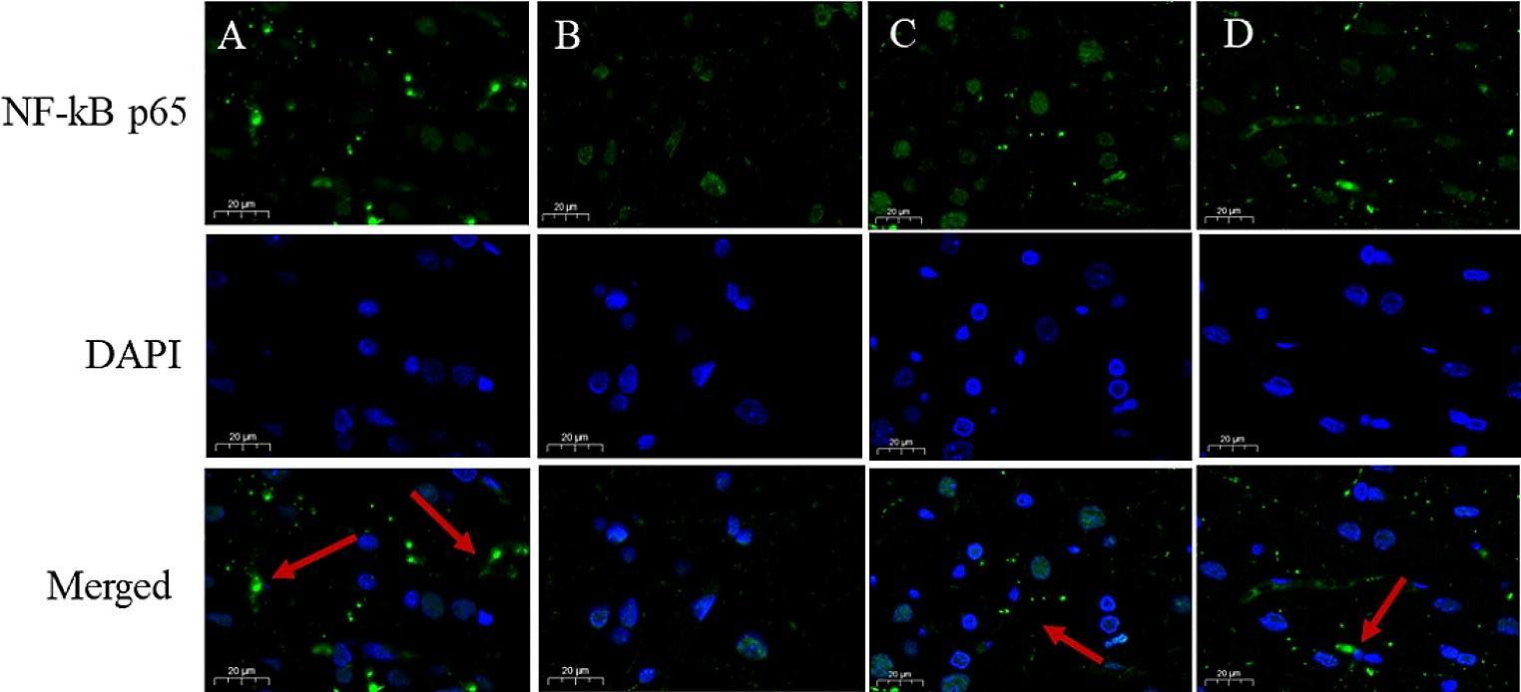
Subcellular localization of NF-κB in rat brain. Representative photographs of IF (NF-κB) were obtained from the (**A**) Control group; (**B**) SS group; (**C**) SS + LGA group; (**D**) SS + HGA group

The MAPK pathway primarily includes JNK, p38, and ERK pathways, and the phosphorylation levels of the three proteins represent their activation. As illustrated in Figs.11-A and 11-B, the phosphorylation levels of JNK and p-38 in the SS group were significantly higher than those in the Control group ( The full uncropped Gels and Blots images in supplementary file Fig. 11). Compared with the SS group, the SS + LGA group could reverse this change and down-regulate the phosphorylation levels of JNK and p-38. More particularly, the high-dose GA group could down-regulate the phosphorylation levels of the two to below the blank group. Unfortunately, there was no significant difference between the groups regarding the ERK pathway. Enrichment of NF-κB within the nucleus could induce cascade transcription of pro-inflammatory cytokines and chemokines (Cheng et al. 2015). The immunofluorescence results of NF-κB (Fig. 12) demonstrated that many NF-κB positive signals were seen outside the nucleus of the cells of the Control group, and there was less distribution in the nucleus. In contrast, almost all NF-κB in the SS group was distributed in the nucleus. After GA administration, the extranuclear distribution of NF-κB was increased in SS + LGA and SS + HGA groups.

## Discussion

In this study, HMGB1 expression was significantly increased in the hippocampal tissue and serum of SD rats with acute poisoning induced by Semen Strychni. We also demonstrate here in that the inhibition of HMGB1 by administering GA alleviated toxic symptoms in rats and suppressed the expression of pro-inflammatory cytokines such as TNF-α, IL-1β, and downstream proteins, including JNK, p38, and NF-κB via the MAPK signaling pathway, which is consistent with the research of Cihan Gur et al. (Gur et al. 2021). In addition, we found that the detoxification effect of GA could be achieved by inhibiting the phosphorylation and release of HMGB1.

Semen Strychni is regarded as a highly toxic herb medicine according to Chinese pharmacopeia. About 10 min after administration with Semen Strychni extract at the dose of 0.2 g/kg, rats in the SS group began to display poisoning symptoms, as reported earlier (Philippe et al. 2004). The hippocampus is a major target of neurotoxic stress response; therefore, we chose the hippocampus for further study. Similar to previous reports, our histopathological findings revealed that Semen Strychni caused organic damage and apoptosis in the hippocampus (Wang et al. 2021; Duan et al. 2022). In addition, according to the results of our experiments, the damage primarily occurred in the CA1 and CA3 regions. The literature suggests that the pathogenesis and central toxicity progression of multiple neurodegenerative diseases are associated with abnormal levels of AChE and MAO (Patlolla A K, Tchounwou 2005; Huot and Fox S H, Brotchie 2016; Nasr and El-demerdash F M, El-nagar W 2016). This experiment showed that the levels of MAO and AChE in the SS group were significantly reduced, indicating that the metabolism of monoamine neurotransmitters and acetylcholine was inhibited, and the treatment with GA could reverse this trend. In addition, nerve damage caused by Semen Strychni can affect the synthesis, storage, and release of other neurotransmitters, causing disorders of various neurotransmitters in the brain, including serotonin, acetylcholine, and dopamine (Shi and Hou 2017; Sun et al. 2018a, b). In summary, Semen Strychni could significantly disrupt the normal levels and functions of neuronal cells and neurotransmitters.

At present, the molecular mechanism of Semen Strychni-induced neurotoxicity is not very clear. Some studies have shown that neurotoxicity induced by chemicals such as sodium fluoride in rats is associated with factors such as neuroinflammation and neuronal apoptosis (Caglayan et al. 2022; Celik and Kucukler 2020; Yıldız and Caglayan 2022). Recently, numerous reports have suggested that HMGB1 as a DAMP is involved in various pathological processes, including stroke, cerebral hemorrhage, inflammatory kidney injury, and heart disease (Liu et al. 2019), which suggested that HMGB1 may be one of the key factors that induced neuroinflammation. Correspondingly, there is a paucity of research on the mechanism whereby GA alleviates the toxicity of SS. In previous studies, GA was commonly used in combination with Semen Strychni to reduce its content and alleviate toxicity (Guo et al. 2018). Accordingly, we used intraperitoneal injection of Semen Strychni and oral administration of GA to avoid their direct reaction. In this study, the distribution of HMGB1 in the hippocampus was determined to explore its role in Semen Strychni poisoning and GA detoxification. According to the immunofluorescence results, the positive signal of HMGB1 in the SS group was concentrated in the cytoplasm, indicating a significant increase in HMGB1 in the cytoplasm. Surprisingly, we also observed the process of HMGB1 release from the nucleus. The application of GA inhibited this process, allowing HMGB1 to remain more in the nucleus. Western blotting was used to further determine the distribution of HMGB1, and the results are consistent with the immunofluorescence results, indicating that high-dose GA could reverse the release process of HMGB1 and reduce the level of cytoplasmic HMGB1 to normal levels. We also performed ELISA analysis of serum HMGB1 levels, and the results demonstrated that serum HMGB1 levels and inflammatory factor levels were significantly elevated in the SS group, and GA reduced these cytokine levels. Based on these results, we preliminarily confirmed that SS could induce HMGB1 release in neurotoxicity.

Studies have demonstrated that phosphorylation is one of the key steps in the release of HMGB1 from the cell, and hyperphosphorylated HMGB1 is more easily translocated from the nucleus to the cytoplasm (Youn and Shin 2006). Studies have reported that PKC can phosphorylate HMGB1 (Kang et al. 2009), while PP2A can dephosphorylate phosphorylated HMGB1 (Taira et al. 2013), thereby reducing HMGB1 nucleocytoplasmic shuttling and subsequent extracellular release. The co-immunoprecipitation results demonstrated that the interaction between PP2Ac and HMGB1 was the weakest in the SS group. Compared with the SS group, the binding of PP2Ac to HMGB1 was increased in the GA group. Simultaneously, the binding of HMGB1 to PKCα in the SS group was enhanced, but this binding could be partially blocked by GA. These results all substantiated the hypothesis that GA may inhibit HMGB1 phosphorylation and release by inhibiting HMGB1 interaction with protein kinases and enhancing its interaction with dephosphorylase.

The MAPK signaling pathway primarily includes extracellular signal-regulated kinase (ERK), c-Jun N-terminal kinase (JNK), and p38. MAPK can be activated by extracellular HMGB1, leading to the inflammatory response. It has been demonstrated that GA as an inhibitor can directly bind to HMGB1 and inhibit extracellular HMGB1 cytokine activity, thereby exerting neuroprotective effects (Sun et al. 2013; Mollica et al. 2007; Tan et al. 2018). Evidence suggests that GA can ameliorate diabetes-induced kidney damage and inflammatory responses by inhibiting the activation of ERK, p38, and NF-κB (Zhang et al. 2017). GA has also been shown to protect against HMGB1-dependent p-JNK/Bax pathway in cardiac ischemia–reperfusion injury (Zhai C L, Zhang M Q, Zhang et al. 2012). We found that GA reversed the increase in the phosphorylation levels of JNK and p38 caused by SS while inhibiting the transfer of NF-κB to the nucleus, alleviating the neurotoxicity of Semen Strychni. In addition to the direct regulation of HMGB1 on its downstream MAPKs and NF-κB pathways (Yardim and GUR 2022; Yardim A, Kandemir F M et al. 2020), studies have found that this pathway is negatively regulated by PP2A and positively regulated by PKC (Nematullah and Hoda M N 2018). Regretfully, it remains unclear whether HMGB1 is involved in the regulation of PP2A and PKC on the downstream pathways. However, activation of MAPKs and NF-κB pathways was certainly blocked by GA.

In summary, we explored the detoxification mechanism of GA by establishing a rat model of acute neurotoxicity induced by Semen Strychni. This study preliminarily demonstrates that Semen Strychni-induced neurotoxicity is partially mediated by HMGB1 and promoted by phosphorylation. GA has the function of inhibiting HMGB1 release, as well as downstream inflammatory pathways, which may be responsible for its alleviation of SS neurotoxicity. Therefore, the potential effect of GA in reducing SS neurotoxicity and its mechanism deserves further study.

## Conclusion

This study successfully established a Semen Strychni neurotoxicity model and confirmed that GA exerted a certain ameliorating effect on the inflammatory response, neuron apoptosis and degeneration, and neurotransmitter metabolism disorder. Furthermore, the key role of HMGB1 in the process of Semen Strychni-induced nerve injury was clarified. On the one hand, GA could inhibit HMGB1 phosphorylation and reduce its transfer to the cytoplasm and extracellular release. On the other hand, it could directly inhibit the extracellular cytokine activity of HMGB1 and the activation of downstream inflammatory pathways, thereby reducing neurotoxicity.

## Data Availability

The used data are confidential.
